# Supplementary material for: Modelling novelty detection in the thalamocortical loop
Source: PLoS Comput Biol. 2023 May 15;19(5):e1009616. doi: 10.1371/journal.pcbi.1009616 (PMC10212104; doi:10.1371/journal.pcbi.1009616)
Supplement: Attachment — Submitted filename: Response to reviewers.pdf [file pcbi.1009616.s003.pdf]

Responses and Changes for PCOMPBIOL-D-21-02007: Modelling novelty detection in the thalamocortical loop

Chao Han, Gwendolyn English, Hannes P. Saal, Giacomo Indiveri, Aditya Gilra, Wolfger von der Behrens, Eleni Vasilaki

---

We would like to sincerely thank the editor-in-chief, associate editor and three anonymous reviewers for the valuable comments. We have thoroughly addressed all comments in this rebuttal letter and revised our manuscript substantially both in writing and by adding new figures. Please note that we have **highlighted** the changed text in red addressing the concerns in our revised manuscript. We are convinced that our revised manuscript has improved significantly and provides now an even stronger model of how the thalamocortical loop can give rise to deviant and novelty signals.

Email addresses: c.han@sheffield.ac.uk (Chao Han), e.vasilaki@sheffield.ac.uk (Eleni Vasilaki)

Responses to Comments from Reviewer 1

Han et al. built a multi-scale recurrent network based on a thalamocortical network model with synaptic depression to simulate the phenomenon of SSA and MMN by rendering adaptation property on those Layer 6 cortical neurons. Their model can explain the novelty detection mechanism in the thalamocortical loop. Meanwhile, this computational model is the first one to delineate the biphasic sensory response in the whisker-related part of S1. This paper shows a logical construct and clear experimental procedure to verify the hypothesis. However, whether other layers especially Layer 5 neurons contribute to the formation of cortical SSA is not incorporated in the current model, which cannot be neglected in the physiological condition. Therefore, some issues should be addressed (see below) before acceptance.

Comment 1.1: "we modelled each barreloid in the VPM nucleus by a cluster of 100 TC cells mediated by a pool of 100 RE cells," May I ask if 100 as the population of cells is a reasonable parameter? Maybe you could cite a reference to explain your choice of population. In addition, I am interested in the effect of the different populations such as 10 or 1000 on your results. Similarly, I am confused if random stimulation to different proportions of TC cells in the two complementary subgroups of each barreloid with sensory input affects your experimental results. I am very pleased to see you try it.

Response 1.1: Thanks for the comment. Our choice of the population size of 100 in each barreloid is based on the real order of the number of neurons in a barreloid of rats that ranges from 100 to 300 neurons (see Table 1 of [1]). The average number of cells in a barreloid is usually even less in mice due to the smaller size of the brain. Therefore the population size used in our model is a biologically plausible parameter. We also tested the effect of different thalamic population sizes (50 and 500 cells) on stimulus-specific adaptation (SSA) and true deviance detection in the early and late responses of our thalamocortical model (see Figure 1 of the rebuttal letter). The strength of SSA and true deviance detection are respectively quantified by the SSA index (SI) and context-specificity index (CSI), whose positive values indicate the existence of the effect (Please see the newly added Data analysis subsection of the revised manuscript for detailed descriptions of the two metrics, lines 628-651, page 26). We found the SI and SCI of late responses varied largely across different orders of the number of thalamic neurons as the rhythmic activity of the thalamic network is sensitive to its network size; for example, the late oscillatory response cannot even be evoked by whisker stimuli

in the case of 500 cells.

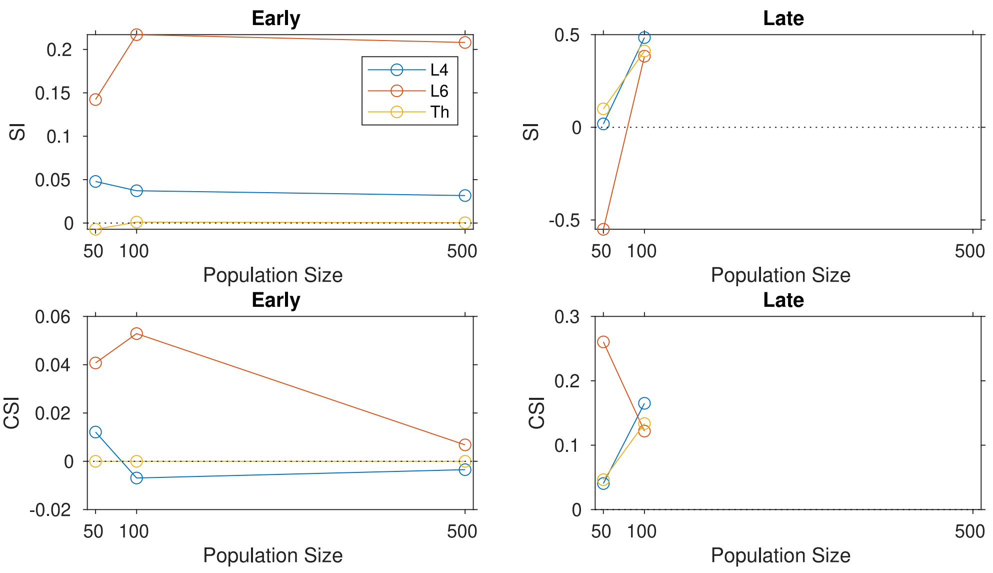

Figure 1: **Effect of thalamic population size on SSA and true deviance detection displayed in early and late responses.** Early and late SIs/CSIs are respectively computed over temporal windows [0, 40] ms and [100, 400] m, where time point 0 denotes the stimulus onset. The absence of SI/SCI values in the case of 500 neurons is due to the failure to trigger thalamic rhythmic bursting activity.

With regard to the second part of the reviewer's question, the reason that we chose the proportion of post-inhibitory bursting TC neurons to all TC neurons $P_{\text{TC}} = 0.6$ is to model the decaying thalamic oscillation by trading off the duration and strength of the thalamic rhythmic bursting evoked by the initial cortical feedback. Figure 2 of the rebuttal letter shows more substantial but less sustained rhythmic firing patterns after the first transient responses, given the increasing rate of post-inhibitory bursting TC cells. In the Material and methods section of the revised manuscript, we added the reference for the thalamic population size (lines 568, page 22) and the above clarification for the proportion of TC cells that we used (lines 583-589, page 23).

Comment 1.2: **In Fig 7 and Fig 8, I am very interested in the CSI of L6 in the late response, although I know the additional late responses can hardly spread into L6. I am very thanks for you if you show me that.**

Response 1.2: As can be seen in Figure 3A of the rebuttal letter, large variances of CSIs of the late L6 responses suggest that late L6 activity cannot exhibit novelty-predicting effects reliably, presumably because secondary responses can hardly spread into L6 from L4. In the revised

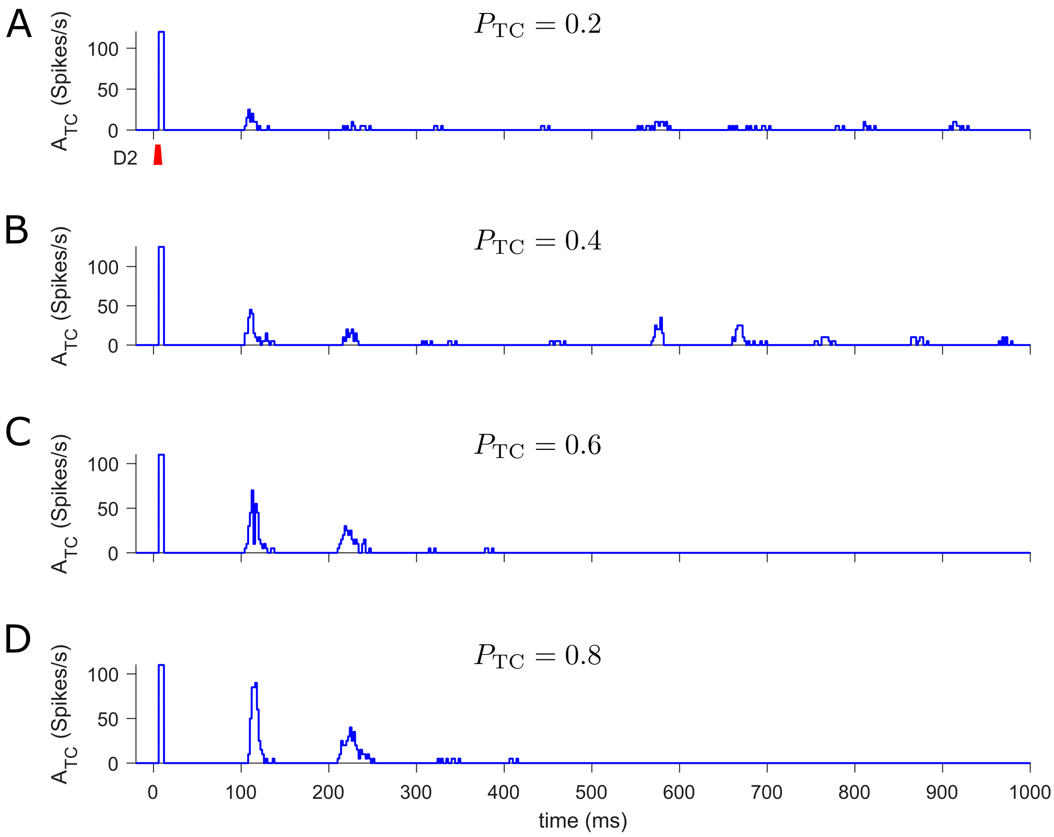

Figure 2: **Response patterns of TC cells in thalamic networks with different proportions of TC cells in the two complementary subgroups of each barreloid.** A brief D2 whsiker deflection evokes different patterns of TC population activity A_{TC} (averaged over all 100 TC neurons) in the principle barreloid, where the proportion of post-inhibitory bursting TC neurons to all TC neurons P_{TC} increases (**A**: $P_{\mathrm{TC}} = 0.2$, **B**: $P_{\mathrm{TC}} = 0.4$, **C**: $P_{\mathrm{TC}} = 0.6$ that is the one used in our model, **D**: $P_{\mathrm{TC}} = 0.8$). The D2 whisker deflection stimulus is marked in red under the first panel.

manuscript, we replaced the old Fig 7 and 8 with the new figure here and removed the related paragraphs describing the old figures (lines 338-339, page 14; page 15).

Comment 1.3: **You maybe explain more about your hypothesis that L2/3 and L5 contribute little to the cortical SSA except citing these papers because this is the basis of your model architecture. For example, you could compare between excitation levels of different layers according to some biological references.**

Response 1.3: The goal of this study was to investigate the role of the thalamocortical loop in detecting novel/deviating stimuli and generating a corresponding deviant signal. It has been

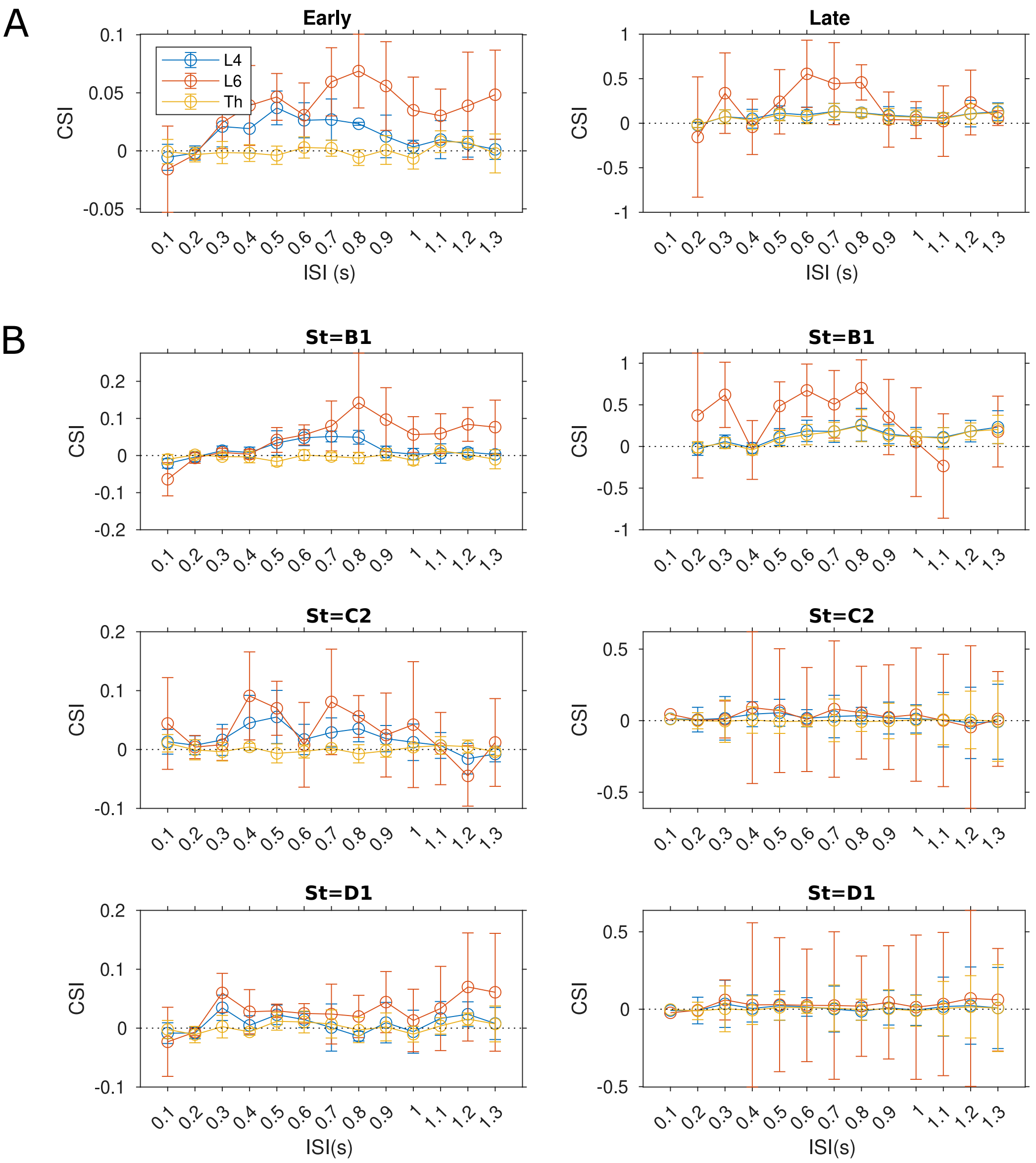

Figure 3: **Dependence of novelty-predicting effects in early and late responses on inter-stimulus intervals (ISI). (A)**Early and late CSIs are respectively computed within time windows of 40 ms after stimulus onset and the remaining time before the onset of the next stimulus. The mean deviant responses in the CSI are averaged over 30 deviants out of 1000 stimuli in each paradigm. **(B)** Similarly, early and late CSIs are computed individually for the C1 deviant occurring right after the B1, C2 and D1 standards in the sequenced condition. The mean responses to each deviant type are averaged over 10 deviants out of 1000 stimuli. Each condition is repeated 5 times with different random seeds to calculate the mean and variant of the CSI values.

proposed before that this loop is a critical player in sensory adaptation (e.g., see Figure 5 in [2] or [3]) and potentially in deviance/novelty detection [4]. Therefore, we limited/constrained our model to the most important "ingredients", meaning VPM, TRN and barrel cortex layers 4 and 6, while leaving out other important structures and layers. Such a minimalistic model nevertheless is helpful for understanding which parts/components are really essential for a certain computation. We emphasize this aspect and limitation of our model in the Results section (lines 109-111, page 4) and Discussion section (lines 369-373, page 16) of the revised manuscript now. We certainly do not want to say that the other cortical layers do not play a role in SSA. We mention now explicitly throughout the revised manuscript that our model is "minimalistic" and that we do not try to emulate the complete cortical circuitry.

Comment 1.4: **Some claims should be properly supported by references, for example: Line 197: "SSA is generally tested in the oddball protocol, where repetitive deflection is applied to 197 one whisker (called the standard), and the sequence is randomly interrupted from time 198 to time by deflection of another whisker (called the deviant)." Line 497: "It has been observed that a cortico-thalamo-cortical loop structure exists in the rodent 497 whisker system." It is suggested that authors check the whole manuscript carefully.**

Response 1.4: We have checked the manuscript thoroughly and added proper references to the claims we made. The references can be found in line 200, page 8 and line 512, page 20 of the revised manuscript.

Responses to Comments from Reviewer 2

The manuscript by Han et al. describes a model for mismatch negativity (MMN). The model consists of mean field models for pools of cortical neurons in layers 4 and 6 (L4 and L6) and simplified Izhikevitch models for interacting excitatory and inhibitory neurons. The key features of the model is that intracortical synapses allow the vertical spread of activity from L4 to L6 and the lateral spread of activity across barrels in L4 and L6. L6 activity recruits thalamic spindle oscillations which give rise to late rhythmic responses in the cortex. The basic idea of the model is that due to synaptic depression of L4 to L6 projections, repeated stimuli do not evoke enough L6 activity to recruit thalamic spindle oscillations (via strong corticothalamic feedback to reticular neurons). A deviant stimulus is able to recruit these spindles due to absence of depression. In a many standards condition, there is less depression of thalamocortical synapses in the control pathway, leading to greater L4 activity and lateral spread of activity, which leads to some synaptic depression that prevents effective recruitment of layer 6 and thalamic spindles.

I find the basic idea (that MMN is driven by synaptic depression limiting the interlaminar spread of activity which in turn limits the recruitment of thalamic spindle activity that could drive late rhythmic responses) potentially interesting. However, I think there are some questions about the robustness of these results (see comment 2), their relationship to what is known about the biology (see comments 1 and 3), which specific aspects of the model are actually necessary (see comment 1), and some apparent inconsistencies (see 4).

Comment 2.1: **I'm a bit confused about the many-standards condition for two reasons. First, does the lateral spread of activity occur mainly via synapses in L4 or L6? It seems like it is in L4 based on Fig. 5B-C. If you had spread of activity mainly through L6 synapses, then you wouldn't get L4 to L6 depression, so this wouldn't work right? So doesn't the model make a strong prediction that MMN depends on lateral connections before L6 (most likely in L2/3)? Does this mean you could you dispense with L6 connections and have the model work just as well? And how well does this line up with the anatomy – my understanding is that there are many more lateral connections in L2/3 (and L5) than L4 or L6. This should be discussed.**

Response 2.1: We thank the reviewer for the comment. First, we would like to clarify that the synaptic depression of intra-column connections in L6 is the primary cause of the generation of deviant-selectivity in the early L6 and late L4 responses, although depression of the thalamocortical

and L4-to-L6 synapses also contributes to expanding the parameter regimes of network model giving rise to SSA.

To understand why the average early response of the L6 network for the oddball deviant is larger than for the many-standards deviant, i.e., true deviance sensitivity in the early L6 response, we need to compare the dynamics of deviant responses of L4 and L6 between the two conditions. For both the many-standards and oddball conditions, the synaptic resources of the deviant column depleted by the cross-whisker activity of the standard(s) in L4 are able to recover to their almost full state when the following stimulus is presented, due to the relatively fast recovery of L4 resources compared with the inter-stimulus interval. Therefore the L6 populations receive comparable inputs from L4 in both conditions. On the other hand, provided a slow recovery of L6 synaptic resources, the activity of the equiprobably distributed standards sometimes is strong enough to propagate into the deviant column in L6 in the many-standards condition, while the repeated oddball standard can hardly do so. Consequently, in L6, the oddball deviant column with complete resources usually evokes stronger responses than the many-standards deviant column with inadequate resources consumed by the activity of the standards, which produces the context-dependent deviance sensitivity. The above clarification is also added in the Results section of the revised manuscript (lines 269-283, lines 301-305, pages 12-13).

As the reviewer mentioned, our network model demonstrates that the vertical spread of population activity from L4 to L6 and lateral spread of activity across barrels in L4 and L6 are mechanistically enough to induce the late deviant-specific response. Since the potential role of L2/3 and L5 in the cortical SSA is still unclear, here we focus on providing a mechanistic explanation of the late response using the minimalistic cortical structure consisting of L4 and L6, which are the two main layers interacting with the thalamus.

Comment 2.2: **A second related issue is about the parameter space and robustness of the model. It seems like the model has been finely tuned so that in the many standards condition activity spreads a bit to other columns and activates their L4→L6 synapses just enough to produce some depression but not so much that you get extensive recruitment of thalamic spindles in these pathways. How robust is this, i.e., can you show some measure of the parameter space and how much you can enhance/weaken the strength of the lateral connections and still get this effect?**

Response 2.2: Thanks to the reviewer for the comment. We tested the sensitivity of both SSA and true deviance detection to the strength of lateral connections within L4, J^1_{L4}, L6, J^1_{L6},

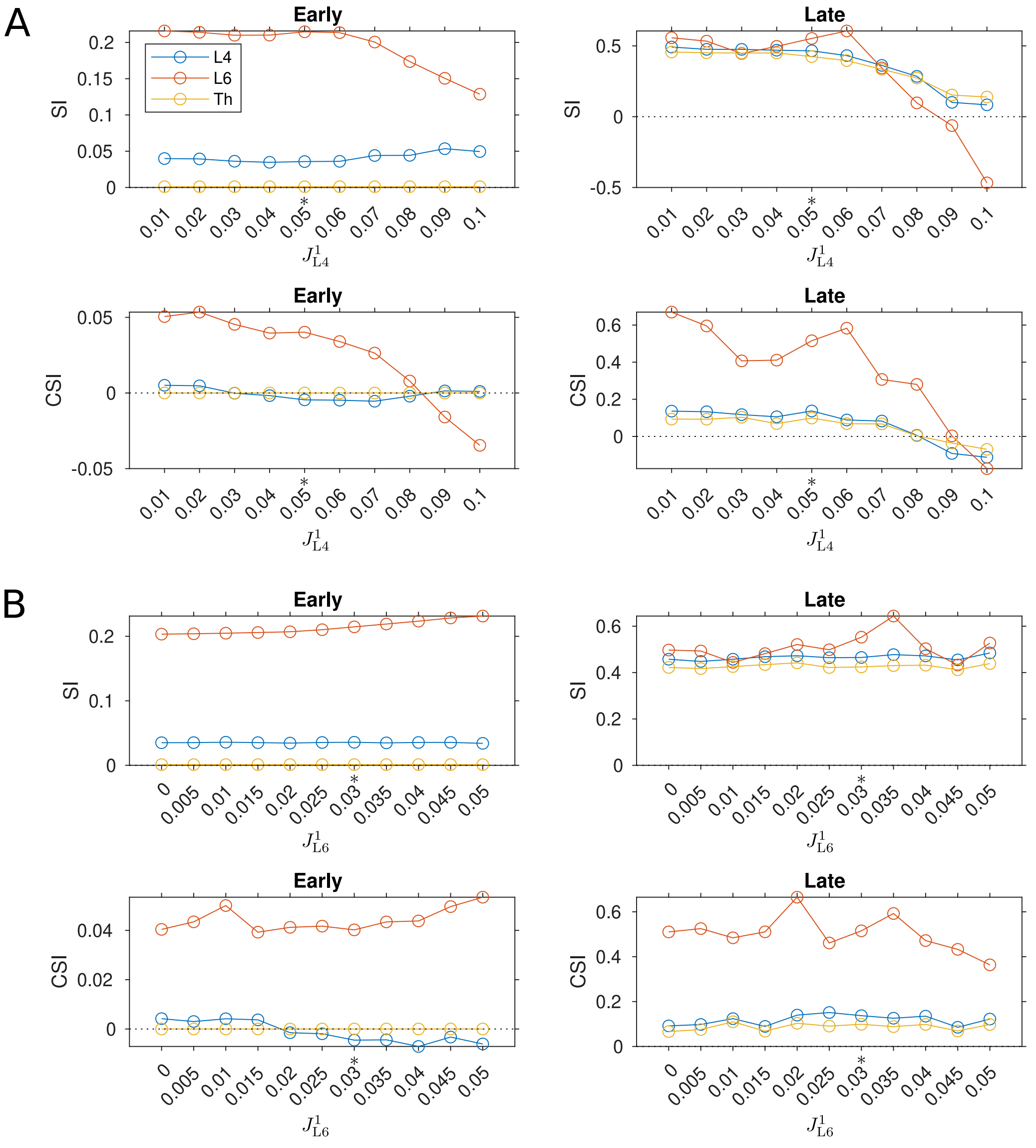

Figure 4: **Sensitivity of SSA and true deviance detection to the lateral cortical connections. (A)** Sensitivity of deviance-detecting effects in early and late responses to lateral connection within L4, J^1_{L4}. **(B)** Sensitivity of deviance-detecting effects to lateral connection within L6, J^1_{L6}. The starred values under the horizontal axis represent the given values in Table 1 of the manuscript. The random seed is fixed for the simulation of each network parameter listed under the horizontal axis.

and the recurrent connections within L6, J^0_{L6}, by varying each of them individually and fixing all the other network parameters in their given values. The effect of SSA and true deviance detection are respectively quantified by the SSA index (SI) and context-specificity index (CSI), whose positive

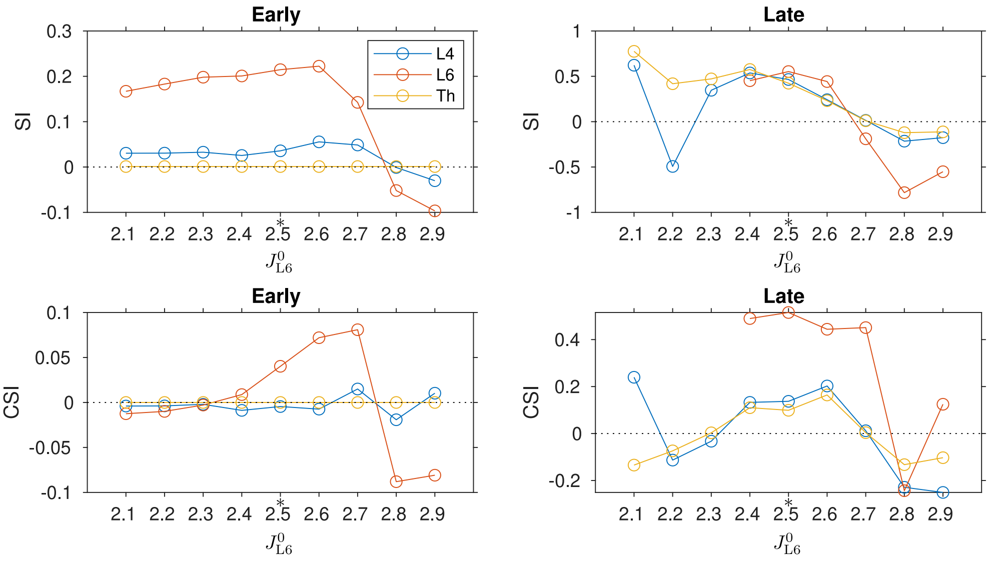

Figure 5: **Sensitivity of SSA and true deviance detection to the recurrent connections wthin L6, J^0_{L6}.** The starred value under the horizontal axis represents the given value in Table 1 of the manuscript.

values indicate the existence of the effect (Please see the newly added Data analysis subsection of the revised manuscript for detailed descriptions of the two metrics, lines 628-651, page 26). As shown in Figure 4A of the rebuttal letter, deviance-detecting effects no longer exist when J^1_{L4} exceeds the critical value of 0.07. On the other hand, deviance-detecting effects are always exhibited within the tested range of J^1_{L6}, even when its value is reduced to 0 (Figure 4B of the rebuttal letter). Finally, the operating range of J^0_{L6} is from 2.5 to 2.6 where the model still demonstrates qualitatively similar deviant behaviours to the experimental observation (Figure 5 of the rebuttal letter). The relatively lower ratio of the operating range of J^0_{L6} to its given values indicates that the deviance-detecting effects in our model are more susceptible to recurrent than lateral cortical connections. We have supplemented the above experiments in the Material and methods section of the revised manuscript (lines 645-661, pages 25-27).

Comment 2.3: **Based on the model, it seems like there should be more robust late responses (corresponding to weak spindle-driven activity) in response to the standard stimulus in the many-standards condition compared to the single-standard (oddball) condition. How well does this line up with experimental observations?**

Response 2.3: The phenomena can be seen in the experiments as well, as shown in Figure

4B of the revised manuscript. Since all the four whiskers are deflected equiprobably in the many-standards condition, the average deviant response should be comparable to any of the standard responses assuming there is no strong response bias toward any of the stimulated whiskers. One can see from Figure 4B of the revised manuscript that the late responses to the many-standards deviant (green curve in the bottom-right panel, ∼10 Hz), which can be a proxy of the standard responses in the many-standards condition, is weaker than to the oddball standard (blue curve in the top-right panel, ∼5 Hz). This response difference observed in the experiment is also captured by our model (compared with the top-right panel of Figure 4C of the revised manuscript).

Comment 2.4: I'm confused by comparing Figs. 4C and 5D. In 4C there is similar late TC activity for the oddball deviant and many-standards deviant. But in 5D there is almost no late TC activity for the many standards deviant. What explains this discrepancy?

Response 2.4: The activity of TC cells evoked by the deviant in both oddball and many-standards conditions in Fig 4C is averaged over 30 presentations of the deviant out of 120 total stimuli. In Fig 5D, we chose two typical examples to demonstrate that the L6 drive is large enough to elicit thalamic rhythmic bursting in the oddball condition, which does not occur in the many-standards conditions. In general, the many-standards deviant is sometimes able to induce the thalamic bursting but not as often as the oddball deviant does, which results in a lower mean TC activity in response to the many-standard deviant than the oddball deviant.

Comment 2.5 Overall I found the figures difficult to decipher. Lots of traces are shown, in many cases different traces corresponding to different things in different panels. This is particularly true for Figure 5 – some panels compare C2 traces for x_{L4} whereas others compare D2 traces for x_{L6}. It would be easier if the same thing was shown consistently, with arrows and boxes to highlight the key points. As it is, it is very hard to keep track of what is going on.

Response 2.5: Thanks the reviewer for the suggestions. We have modified some of the figures in the revised manuscript to make them easier to digest, which are listed as follows:

1. We matched the legends of Fig 4B to that of Figure 4C and illustrated the flow of the excitatory activity inducing the late context-dependent deviant response via a flowchart in Fig 4C (page 11 of the revised manuscript).
2. We removed panel D of the standard responses in Fig 5 to consistently show the trace of

deviant responses across L4, L6 and thalamus (page 12 of the revised manuscript).

Responses to Comments from Reviewer 3

Stimulus specific adaptation (SSA) is a phenomenon where responses to deviant stimuli are larger than the responses to non-deviant stimuli. The effect was mostly described in the auditory cortex, but was found also in the whisker somatosensory cortex. The manuscript describes a mechanistic explanation to the effect: neurons in cortical layer 6 adapt, via synaptic depression, specifically to a frequently presented stimulus, resulting in reduced population activity when compared with the population activity evoked by a rare stimulus. This difference is projected from cortex to thalamus and generate decaying spindle oscillations. As a result, layer 4 neuron in cortex display late responses to rare stimuli.

Comment 3.1: **The mechanism presented in the model is interesting, but suffers from substantial shortcomings. Spindle oscillations are generated during sleep or anesthesia, when thalamic neurons are hyperpolarized and function in a "bursting mode", during which they possess post-inhibitory rebound and fire a calcium spike when released from hyperpolarization. In awake animals, however, bursting model is rarely seen and thalamic neurons fire tonically in response to excitatory stimulation. Therefore, a mechanism based on activity during sleep cannot explain sensory responses in behaving animals.**

Response 3.1: We would like to thank the reviewer for this and the following Comment 3.2 as it concerns a very important issue that was apparently not sufficiently clearly written. We did not strive to model sleep spindles in this study! Instead, we utilized an integrate-and-fire model (such as the Izhikevich model) to generate thalamic bursting spiking dynamics, which reproduces the spiking patterns of spindles modelled by a more detailed Hodgkin–Huxley model published by Destexhe et al. [5]. This was not meant to model actual sleep spindles but is a tool to build a phenomenological model of thalamic activity. This strategy to utilize Destexhe's model [5], or at least some of its parameters, has been used before to model other phenomena with thalamic involvement, such as pathogenesis in childhood absence epilepsy [6], or more generally to model "[...] *rhythmogenesis, involving post-inhibitory rebound excitation in the TC neurons*" [7]. In order to make this more transparent, we changed or removed the references to spindles throughout the manuscript, except when explaining the model in the Discussion section (lines 392-401, pages 16-17), as this is clearly not the goal of our study!

The other directly related issue raised by the reviewer is if thalamic neurons exhibit bursting

firing patterns. The firing rate and pattern depend on the exact whisker stimulation parameters (acceleration, amplitude, etc.) and condition (active or passive, repetitive, etc.), which have to be considered for estimating how realistic the model is. We think that our model of the VPM firing induced by passive whisker stimulation is actually correct for both, the firing rate (first response ∼100 spikes/s, later responses ∼40 spikes/s) and the firing pattern (some bursting, some not) (see Figure 2C and D of the revised manuscript).

In the study cited by the reviewer [8], the VPM firing pattern was recorded during active touch, when the mouse moved the whisker towards a pole to detect it. While the average response/touch in [8] is indeed just 0.6 spikes, Figure 1D of [8] shows at least one thalamic neuron (VPM No. 14.3.244) that seems to exhibit a bursting firing pattern in many (but not all) trials, which is how we modelled the VPM firing pattern as well (even though most/all of our thalamic neurons exhibited some bursting). In some trials, our stimulated VPM neurons are not firing at all and in particular, later in the stimulation sequence after multiple stimuli have been presented (Figure 3D of the revised manuscript, e.g., look at the time points 1-6 seconds). In supplementary Figure 1B of [8] ("Extracellular recordings in VPM during whisker-based object localization"), the authors show that passive stimulation of whisker results in a firing of thalamic units of at least 200 spikes/s, which actually matches the order of magnitude of the spike rate we model in the VPM neurons (initially ∼100 spikes/s, later ∼40 spikes/s).

Other studies have reported similar bursting thalamic activity, in particular when the whisker stimuli were applied passively, as shown for example in Figure 1E of [9] and in Figure 3A of [3], or spontaneously in awake animals as shown in Figure 1A of [10] and Figure 1C of [11]. Therefore, we believe our model is based on realistic thalamic spiking patterns and firing rates, which have been reported many times before for different conditions and species. We clarified that only passive stimulation of the whiskers is considered in our case in the Discussion section of the revised manuscript (lines 402-406, page 17).

Comment 3.2: Thalamic neurons in the model burst in response to touch (Fig. 2D). In reality, they fire, on average, less than one spike per touch (0.6 spikes/touch; Yu et al., Nat. Naurosci. 12:1647, 2016).

Response 3.2: We thank the reviewer for bringing up this point and giving us the opportunity to elaborate it in more detail as this is a critical aspect of the model. Since we think this comment is directly related to the previous one, we provide an answer to both comments above (Response 3.1).

Comment 3.3: The thalamic model display decaying spindle oscillations. To achieve them, parameters of thalamic connectivity should be fine-tuned. Otherwise, one will not obtain any oscillations or obtained sustained oscillations. The model does not include excitatory neurons in cortex. Without them, epileptic-like pulses propagate in cortex. It is not clear what stop them in the model.

Response 3.3: We assume the reviewer means "inhibitory" neurons instead of "excitatory". Indeed, parameters of thalamic neurons and their connectivity strength need to be finely tuned to demonstrate the decaying rhythmic bursting behavior. In order to understand the mechanisms that mediate the termination of the rhythmic activity in our model, we first illustrate in the phase portrait of the Izhikevich model how the post-inhibitory bursts of the thalamocortical (TC) cells can be triggered by the activation of reticular (RE) cells (Figure 6 of the rebuttal leter). A brief, weak negative current pulse pushes the states of a TC cell just outside its attraction basin but in the vicinity of the attractor, where the vector field is relatively small. The membrane potential increases slowly and steadily across the attractor neighborhood and fires a burst of spikes before entering the attraction domain (see Figure 6B). Hence the spike burst is induced with a long latency.

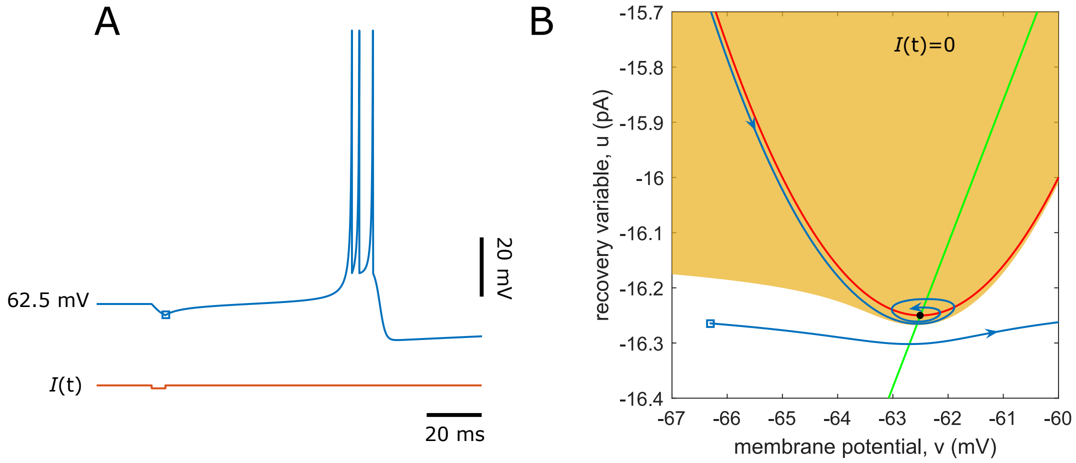

Figure 6: **Post-inhibitory bursts in a TC cell. (A)** A burst of spikes with significant latency is evoked by an inhibitory current pulse with 1 pA amplitude lasting 5 ms that approximates a transient inhibitory postsynaptic current of RE cells modulated by $GABA_A$ neurotransmitters. The membrane potential value immediately after the release from inhibition is denoted in the open square. **(B)** The phase portrait of the Izhikevich model with parameters used in the first subpopulation of TC cells when $I(t) = 0$. The intersection of the v-nullcline (red) and u-nullcline (green) is a stable focus, whose attraction basin is marked in orange. The trajectory initiated at the states right after the removal of inhibitory input (open square, same as in (A)) slowly goes through the neighborhood of the equilibrium before accelerating towards the voltage threshold of the spike peak. After setting off several spikes, the states are reset to the attraction domain and approach along the v-nuclline to the equilibrium (resting potential).

Furthermore, Figure 7 demonstrates how rhythmic bursting initiated by cortical feedback fades away in a thalamic circuit of 20 TC and 20 RE neurons. The TC cells that receive temporary excitatory corticothalamic inputs are driven to a depolarized level that prevents the post-inhibitory bursts (see Figure 7 of the rebuttal letter). In contrast, most of the TC cells that have not received the corticothalamic feedback are capable of initiating the rebound burst in the first round of oscillation, induced by the activity of RE cells after the onset of the corticothalamic stimulation. In the second bursting cycle, some of the TC cells that are silent in the first cycle fire, but the activity of their coupled RE cells are weaker than that in the first round. As a result, very few TC cells are triggered by the reduced RE activity in the third round, and after that, the inhibition from RE cells is not strong enough to elicit any rebound burst in the TC cells, which terminates the rhythmic bursting.

The rhythmic activity initiated in the stimulated barreloid of the VPM only induces inadequate cortical responses in the principal barrel and cannot propagate to the surrounding barrels due to the activation threshold of the cortical neurons.

We added the above analysis and interpretation in the Material and methods section of the revised manuscript (lines 618-644, pages 24-25).

Comment 3.4: **The authors emphasize the small subset of neurons in the granular layer with a deviant-specific late response, occurring roughly 200 ms after stimulus offset, found experimentally in ref. 8. These neurons display late response even to non-deviant stimuli (Fig. 4B), although less oscillatory than the response to the deviant stimulus. In the model, there is late response in thalamus even for a standard stimulus (Fig. 4C), that is also weaker than the response for the deviant stimulus. This means that "spindle-like" oscillations are generated even in response to standard stimuli. In reality, rodent whisk in about 10 Hz (rats) – 15 Hz (mice). This means that responses to subsequent touch events will be "polluted" by oscillations generated in response to the first touch. This will make the responses to consecutive touch events complicated, and, as far as I know, has never been reported.**

Response 3.4: In our case the whisker deflection frequency is 1 Hz, so the thalamocortical (spindle) oscillation has time to fade away before the onset of the next stimulus (see Fig 3D of the revised manuscript). When the animal is in an "active touch" state (e.g., when detecting an object in the dark), it is indeed moving its whiskers at 10-15 Hz, which could potentially interfere with detecting any deviant-related late activity and where one would have to consider the input from the motor cortex into the barrel cortex as well. However, we are modelling a passive stimulation

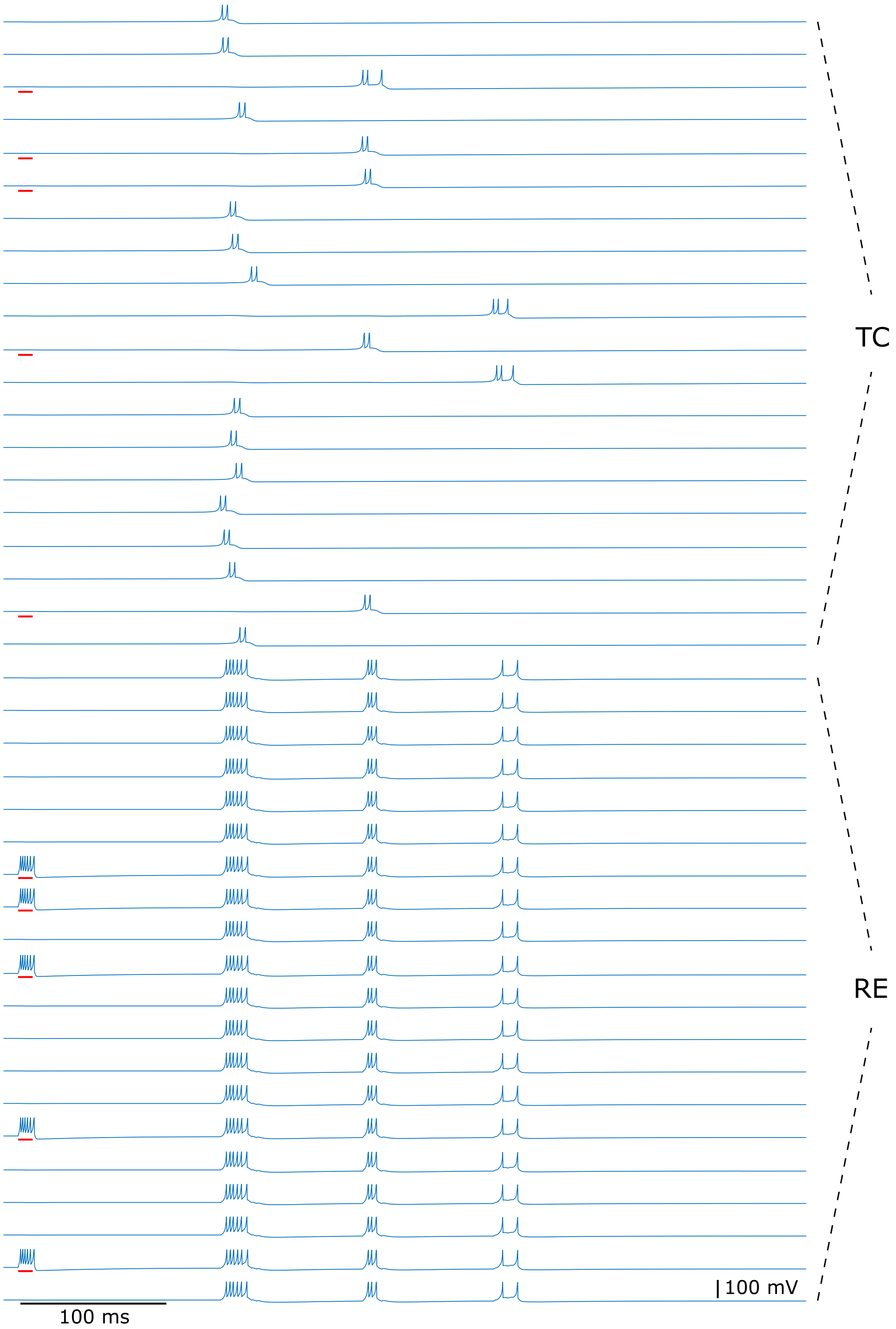

Figure 7: **Rhythmic bursting evoked by cortical discharges in a thalamic circuit of 20 TC and 20 RE cells.** Red bars under the voltage traces mark the initial cortical stimulation that is randomly distributed over 25% of TC and RE cells.

where the subject is not performing an active touch but is just passively exposed (e.g., similar to a situation when the animal is running along a wall). During active whisking, both the barrel cortex and thalamus change their activity significantly and are in a feedback loop with the whisker motor cortex (e.g., see the review [12]). We want to point out that SSA/deviant paradigms generally use carefully controlled passive stimulation and that it is an open question whether the results obtained in our paper might generalize to the active case.

Comment 3.5: **Fig.6 shows that the response to sequenced deviant is almost the same as the response to randomize deviant. I do not understand what the authors tried to say by presenting this figure, and why they emphasized the tiny difference between the two responses.**

Response 3.5: This is an experiment that we actually performed in-vivo in the somatosensory system of the mouse, which is currently under revision in another journal as well. We used this sequence to compare the behaviour of our model with other data and conditions to get a better understanding of how well our model actually captures the biology even for new and different stimulation sequences. What is interesting here is that our model captures, even though with a rather small effect, the differences between two stimulation conditions that are very similar and which only differ in the temporal domain. For a true deviance detection in the nervous system, one would expect different signals for these two conditions nevertheless, and this is exactly what we see in our new experimental data and our model. We hope to publish both manuscripts more or less simultaneously so that they complement each other.

The above clarification was supplemented in the Method section of the revised manuscript (lines 322-327, page 13).

References

[1] H. S. Meyer, R. Egger, J. M. Guest, R. Foerster, S. Reissl, M. Oberlaender, Cellular organization of cortical barrel columns is whisker-specific, Proceedings of the national academy of sciences 110 (47) (2013) 19113–19118.

[2] C. J. Whitmire, G. B. Stanley, Rapid sensory adaptation redux: a circuit perspective, Neuron 92 (2) (2016) 298–315.

[3] R. A. Mease, P. Krieger, A. Groh, Cortical control of adaptation and sensory relay mode in the thalamus, Proceedings of the National Academy of Sciences 111 (18) (2014) 6798–6803.

[4] P. Lakatos, M. N. O'Connell, A. Barczak, T. McGinnis, S. Neymotin, C. E. Schroeder, J. F. Smiley, D. C. Javitt, The thalamocortical circuit of auditory mismatch negativity, Biological psychiatry 87 (8) (2020) 770–780.

[5] A. Destexhe, D. Contreras, M. Steriade, Mechanisms underlying the synchronizing action of corticothalamic feedback through inhibition of thalamic relay cells, Journal of neurophysiology 79 (2) (1998) 999–1016.

[6] A. T. Knox, T. Glauser, J. Tenney, W. W. Lytton, K. Holland, Modeling pathogenesis and treatment response in childhood absence epilepsy, Epilepsia 59 (1) (2018) 135–145.

[7] G. M. Shepherd, N. Yamawaki, Untangling the cortico-thalamo-cortical loop: cellular pieces of a knotty circuit puzzle, Nature Reviews Neuroscience 22 (7) (2021) 389–406.

[8] J. Yu, D. A. Gutnisky, S. A. Hires, K. Svoboda, Layer 4 fast-spiking interneurons filter thalamocortical signals during active somatosensation, Nature neuroscience 19 (12) (2016) 1647–1657.

[9] C. J. Whitmire, C. Waiblinger, C. Schwarz, G. B. Stanley, Information coding through adaptive gating of synchronized thalamic bursting, Cell reports 14 (4) (2016) 795–807.

[10] H. A. Swadlow, A. G. Gusev, The impact of'bursting'thalamic impulses at a neocortical synapse, Nature neuroscience 4 (4) (2001) 402–408.

[11] E. E. Fanselow, K. Sameshima, L. A. Baccala, M. A. Nicolelis, Thalamic bursting in rats during different awake behavioral states, Proceedings of the National Academy of Sciences 98 (26) (2001) 15330–15335.

[12] C. C. Petersen, Sensorimotor processing in the rodent barrel cortex, Nature Reviews Neuroscience 20 (9) (2019) 533–546.
