# Supplementary material for: Modelling novelty detection in the thalamocortical loop
Source: PLoS Comput Biol. 2023 May 15;19(5):e1009616. doi: 10.1371/journal.pcbi.1009616 (PMC10212104; doi:10.1371/journal.pcbi.1009616)
Supplement: Attachment — Submitted filename: Response to reviewers.pdf [file pcbi.1009616.s004.pdf]

Responses and Changes for PCOMPBIOL-D-21-02007:
Modelling novelty detection in the thalamocortical loop

Chao Han, Gwendolyn English, Hannes P. Saal, Giacomo Indiveri, Aditya Gilra, Wolfger von der Behrens, Eleni Vasilaki

---

We would like to sincerely thank the editor-in-chief, associate editor and three anonymous reviewers for the valuable comments. We have thoroughly addressed all comments in this rebuttal letter and revised our manuscript substantially both in writing and by adding new figures. Please note that we have **highlighted** the changed text in red addressing the concerns in our revised manuscript.

Editor Comment 1: Notably, the questions from reviewer 1 must be answered clearly and conclusively. This reviewer questions how realistic are the extensive L6 connections - is there conclusive evidence for them in the cortex?

Response: As explained in our response to reviewer comment 2.1 (below), there are well-described cortico-cortical connections in layer 6 of the rat barrel cortex, for both layer 6a and 6b. Narayanan et al. [2015] describe the cortico-cortical projection pattern of layer 6 neurons of the rat barrel cortex (which is the basis of our model as well) in some detail (e.g. see Fig. 8 there). Marx and Feldmeyer [2013] provide a detailed description of projections in layer 6b of the rat barrel cortex. Therefore, we are confident that our assumption of the lateral projects in layer 6 is biologically justified.

Editor Comment 2: Second question is about results presented in figure 4C and 5D - these issues must be cleared up.

Response: In our answer to reviewer comment 2.2, we clarify that the two selected deviant responses in Fig. 5D of the manuscript are only the extreme cases where late responses are almost exclusively elicited by the oddball deviant. However, overall the responses are very variable, so the two examples shown are not particularly reflective of the mean. We apologize for our mistake of using the wrong word "typical"; instead, the two extreme cases that we select to demonstrate are "atypical". We add an extra Supplementary figure to illustrate the other variants of deviant responses and provide more detailed explanations in our response to the reviewer and in the related paragraph of the manuscript (lines 314-321) to clear up any confusion.

Editor Comment 3: Issues raised by reviewer 2 are also quite serious and must be addressed fully. Notably - the reviewer questions how realistic is the TC bursting mode in vivo non-anesthetized state? Seems like this is a crucial point for the validity of the model.

Response: In our response to reviewer comment 3.1 and in the discussion section of the manuscript, we provide a detailed answer why we believe that our model is indeed relevant for both the awake and the anesthetized condition as stimulus-specific adaptation and mismatch negativity have been reported under both conditions. This concern is now addressed in a new paragraph in lines 370-383. In addition, we would

Email addresses: c.han@sheffield.ac.uk (Chao Han), e.vasilaki@sheffield.ac.uk (Eleni Vasilaki)

like to highlight the paragraph in the discussion section where the issue of the TC bursting mode is discussed (lines 431-439): *"We also want to point out that SSA paradigms generally use carefully controlled passive stimulation rather than active touch (e.g., detecting an object in the dark). Many studies have reported bursting activity in the thalamus similar to our model, when whisker stimuli were applied passively both in anesthetized [76, 77] and awake animals [76, 78], and spontaneously in awake animals as well [79, 80]. While post-inhibitory rebound bursting activity of thalamic neurons is characteristic for the unconscious state, it can be observed in the auditory thalamus of awake, passively listening animals as well in the form of a post-stimulus burst [81], even though at longer stimulus durations than used in the model presented here."* In addition to these references demonstrating the existence of a bursting mode in the whisker thalamus (VPM) in anesthetized and awake animals, more broadly it *"[...] has been posited that regulation of burst firing in the thalamus may serve as a dynamic gating mechanism for controlling information flow to cortex [...]"* (from [Whitmire et al., 2016]). Therefore, we are convinced that our model of the TC bursting has a well-supported biological basis and is indeed relevant for both the anesthetized and the awake condition, making our study relevant for a broad readership interested in sensory processing in the thalamocortical loop.

Editor Comment 3: Realism of the model without cortical inhibitory neurons must be also cleared up - notably the regimes for the SSA and run-away excitation. Furthermore, authors must address the doubts on the robustness of the model w.r.t. parameter choices - e.g. the strength thalamus-cortex coupling etc.

Response: As explained in our response to review comment 3.2, our model employs synaptic depression as a surrogate for inhibitory neurons to prevent overexcitation in the cortex. We show the regimes for SSA and runaway excitation by varying the recovery time of depressing synapses in the cortex. We also test the robustness of SSA in our model with respect to the strength of the intra-cortical, cortico-thalamic and intra-thalamic connection. We report different regimes in which sustained, decaying and absent oscillations in thalamic responses appear respectively (see our response to comment 3.3).

Responses to Comments from Reviewer 2

Comment 2.1: In my original comment 1, I asked "does the lateral spread of activity occur mainly via synapses in L4 or L6... doesn't the model make a strong prediction that MMN depends on lateral connections before L6?" I also asked about how this lines up with what is known about the existence of lateral connections in different layers. In response, the authors state that the depression of intra-column L6 synapses is the primary cause of deviant-selectivity selectivity. They go on to state that the recovery of synaptic resources in L4 means that the input to L6 is similar in the many standards and oddball deviant conditions, resulting in similar L6 activation in both of these cases (and weaker activation in the oddball standard condition). They then state that there is sometimes propagation into the deviant column in the many standards condition but not in the oddball standard condition, which causes depletion of L6 resources in the many standards case. Based on this, it sounds like the model requires lateral connections in L6 to produce a difference between the many standards and oddball deviant conditions. However, I am still unclear on how realistic this is – there are known to be extensive lateral connections in L2/3 and L5, but is this the case for L6, as the model seems to require.

Response 2.1: We thank the reviewer for the comment. Such cortico-cortical connections are known to exist in layer 6 of the rat barrel cortex. They are described in some detail in the extensive study of [Narayanan et al., 2015], which states that "Unpatterned horizontal projections (10% of IC-unit), comprised cell types that were transcolumnar along the row and arc (i.e., primarily L5tt and L6cc)". We have now added a sentence to the results section where we summarize the circuit assumptions of our model (see lines 116-119): "Additionally, we assume lateral cortico-cortical connections in layer 6 as they have been demonstrated in the rat barrel cortex [Narayanan et al., 2015] and even in layer 6b [Marx and Feldmeyer, 2013]. These projections are hypothesized to give rise to the fast horizontal spread of single whisker-evoked excitation."

Comment 2.2: In my original comment 4, I asked about the reason for the difference between Fig. 4C (in which the late response to the many standards deviant is similar to the late response to the oddball deviant) and 5D (in which there is very little late response to the many standards deviant). The authors respond by stating that Fig. 4C is an average whereas Fig. 5D is a "typical example." But it seems that the example in Fig. 5D is not typical of the average response shown in Fig. 4C? Furthermore the average late response of the model to the many standards deviant shown in Fig. 4C seems very different from the experimentally measured late response to the many standards deviant shown in the bottom row of Fig. 4B (in which the late response to the many standards deviant is very weak making it different from Fig. 4C but also has a different timecourse compared Fig. 5C). These discrepancies strike me as problematic and must be clearly addressed.

Response 2.2: Thanks to the reviewer for the comment. We want to clarify that the two selected deviant responses in Fig. 5D of the manuscript are only the extreme cases where late responses are almost exclusively elicited by the oddball deviant. However, overall the responses are very variable, so the two

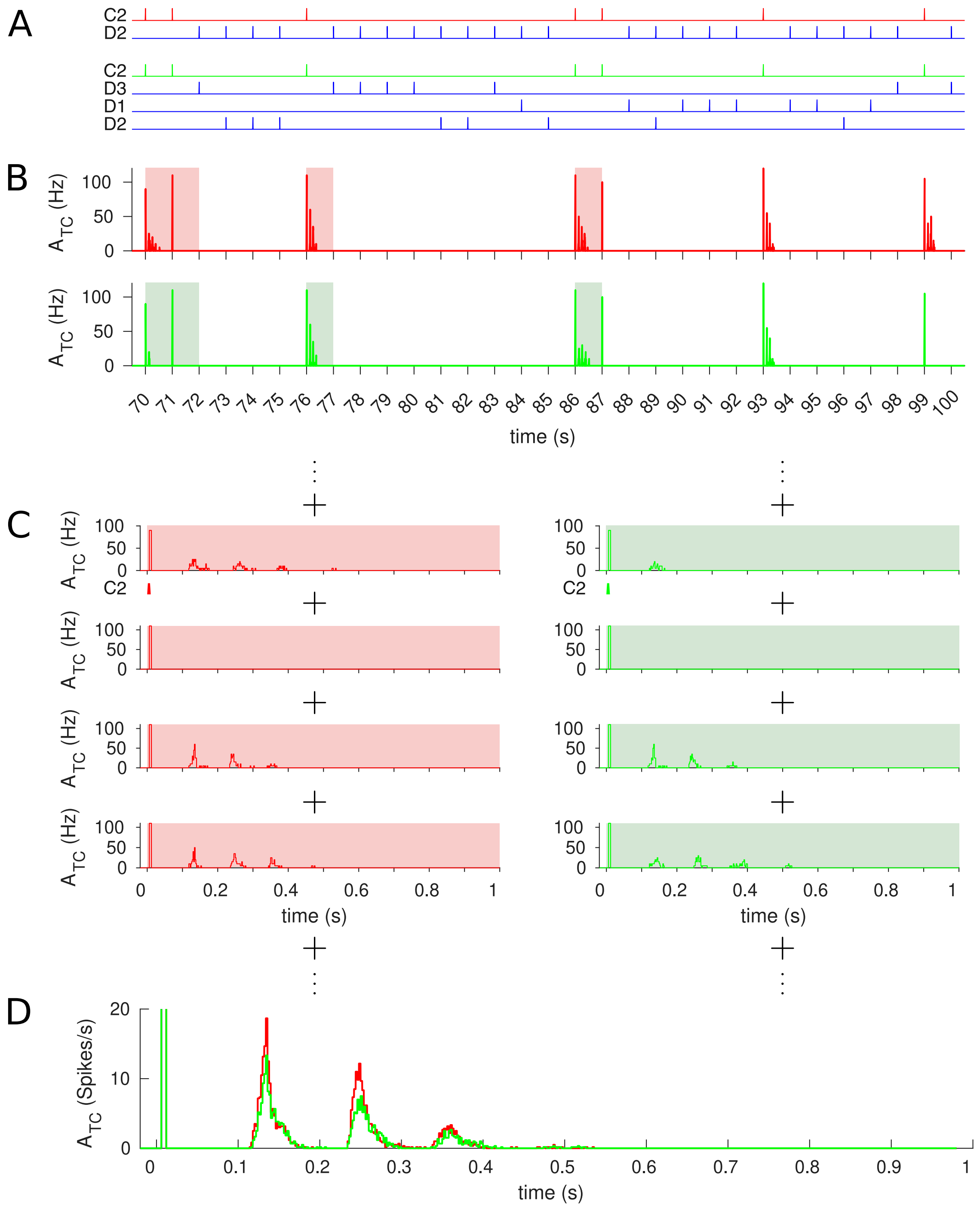

Figure 1: **Population activity of TC cells in response to deviant stimuli in oddball and many-standards conditions.** **(A)** Illustration of oddball (top) and many-standards (bottom) protocols. The deviant stimuli to the C2 whisker are highlighted in red in the oddball condition and green in the many-standards condition. **(B)** Temporal profiles of population activity of TC cells in C2 barreloid evoked by the oddball deviant (red) and many-standards deviant (green). **(C)** Magnified deviant responses in the shaded area denoted in **(B)**. The stimulus duration is marked below the responses in the first row. **(D)** Average TC responses in C2 barreloid over inter-stimulus intervals to the oddball deviant (red) and many-standards deviant (green).

examples shown are not particularly reflective of the mean. We apologize for our mistake of using the wrong word "typical"; instead, the two extreme cases that we select to demonstrate are "atypical". As shown in Fig. 1B of the rebuttal letter, the many-standards deviant can induce late responses as well, although generally not as substantial as the oddball deviant. Therefore the average late responses to the oddball many-standards deviant are weaker than the oddball deviant (Fig. 1C). We have added Fig. 1 as Supplementary Figure S1 of

the manuscript and the above explanations in the related paragraph (lines 314-321). Simulation data for the complete sequences of oddball and many-standard deviant responses and a script for plotting Figure S1 in the manuscript have been uploaded to `https://figshare.com/articles/dataset/Simulation_Results/21614145` (see README.txt for instructions). The codes for all simulations and figures in the manuscript can be found at `https://github.com/ChaoHan-UoS/SomatosensorySSA_Model`.

Regarding the comparison with experimental data, we would like to emphasize that the late responses observed by Musall et al. [2017] were actually quite variable between the different units (e.g. see Fig. 4G in [Musall et al., 2017]). However, the actually recorded many-standards deviant response for single units/neurons and the population local field potential signal (Fig. 5B and C in [Musall et al., 2017], respectively) seems to be rather a scaled-down version of the oddball deviant, as we observe it on our model as well. We would like to emphasize that the data shown in Figs. 5A, B and C of [Musall et al., 2017] are pooled data from many recordings and therefore, different oscillatory response patterns (as shown in Fig. 4G of [Musall et al., 2017]) may be averaged out. We emphasize this aspect now in the results section (lines 267-269) saying "The late responses are only found in a subset of recordings mainly confined to the L4, but with slightly different oscillatory firing patterns in individual single neurons which can result in averaging out these oscillations in the population signal."

Responses to Comments from Reviewer 3

Comment 3.1: **The authors responded to my previous comments. A major source of confusion is the lack of distinction between healthy, awake behavior and cases like anesthesia, absence epilepsy and sleep. Generally, thalamic neurons function in the spiking mode during awake behavior, although there may be certain cases in which some neurons – but not the majority – respond in a burst to stimuli. During some forms of anesthesia, weak sleep and (maybe) absence seizure, the bursting mode is abundant. The authors present SSA as a property of sensory systems, and relate it to mismatch negativity (MMN) that is observed in humans. In contrast, the authors explain SSA using a mechanism based post-inhibitory rebound in TC neurons, which is a hallmark of anesthetized, unconscious states. Post-inhibitory rebound occurs when a TC neuron is hyperpolarized, such as during sleep or anesthesia. For this reason, the authors obtain periodic activity in the frequency of about 10 Hz in response to some stimuli (Fig. 3). They present experimental results from Musall et al. (Fig. 4B), but these experiments were carried out on anesthetized animals. Whether or not a model that is valid for anesthetized animals and aims to explain sensation is interesting or not is a matter of editorial decision. If this manuscript is published, the distinction between awake and anesthetized states should be clearly written. In addition to this issue, there are several other shortcomings in the article.**

Response 3.1: Thanks to the reviewer for raising these critical points which obviously are of high relevance to the broader readership and in particular the large field studying mismatch negativity in human subjects. There are a number of points in this comment that we would like to address separately:

1) **"The distinction between awake and anesthetized states should be clearly written."** In the author summary of the manuscript, we explicitly state now that the physiological basis of this study was in the anesthetized condition: "Here, we developed a minimalistic thalamocortical network model that accounts for novelty detection and reproduces physiologically observed neural response patterns in the anesthetized somatosensory cortex." Furthermore, we have added a paragraph to the discussion section (lines 370-383) where we emphasize that the model was built on data from anesthetized animals. At the same time, we discuss that stimulus-specific adaptation and deviance detection is robustly observed in both awake and anesthetized animals and that in humans mismatch negativity is similarly reported in different brain states, including sleeping, comatose and anesthetized subjects. These statements are supported by a large body of literature (from which we only can cite a small fraction due to space limitations) and apply to different sensory modalities and species.

2) **"A major source of confusion is the lack of distinction between healthy, awake behavior and cases like anesthesia, absence epilepsy and sleep. Generally, thalamic neurons function in the spiking mode during awake behavior, although there may be certain cases in which some neurons – but not the majority – respond in a burst to stimuli. During some forms of anesthesia, weak sleep and (maybe) absence seizure, the bursting mode is abundant."** Another critical aspect raised by the reviewer is the bursting of neurons in the somatosensory thalamus. In the discussion section we specifically address this point and cite references that demonstrate bursting in the

whisker thalamus both in the anesthetized and the awake animal (lines 431-435): "We also want to point out that SSA paradigms generally use carefully controlled passive stimulation rather than active touch (e.g., detecting an object in the dark). Many studies have reported bursting activity in the thalamus similar to our model when whisker stimuli were applied passively both in anesthetized [Whitmire et al., 2016, Mease et al., 2014] and awake animals [Whitmire et al., 2016, Wright et al., 2021], and spontaneously in awake animals as well [Swadlow and Gusev, 2001, Fanselow et al., 2001].". We agree with the referee that the bursting of thalamic neurons in the thalamus is most likely a rare event. However, as shown in Fig. 3D, our modelled thalamic response is more often not bursting than bursting and when doing so most prominently in response to the rare deviant stimulus. We further reported the average percentages of thalamic neurons that are bursting in a trial and the average percentages of trials in which a given thalamic neuron is bursting (lines 238-243): "On average, over trials, 25% of TC neurons in the deviant column (C2) respond in bursts to a deviant stimulus. In contrast, 10% of TC neurons in the standard column (D2) burst in response to a standard stimulus. Besides, a given bursting TC neuron in C2 (Neuron Index 210 in Fig. 3D) bursts in 46% of trials of deviant stimuli, while another bursting TC neuron in D2 (Neuron Index 10) bursts in 17% of trials of standard stimuli." Additionally, the nature of the thalamic bursting activity assumed in our model is far from that observed in any pathological state such as epilepsy, because it ceases after stimulus presentation and is not self-sustaining. Therefore, we consider our model to be potentially relevant for awake subjects as well and hope the referee and editor agree with this assessment.

3) **"In contrast, the authors explain SSA using a mechanism based post-inhibitory rebound in TC neurons, which is a hallmark of anesthetized, unconscious states. Post-inhibitory rebound occurs when a TC neuron is hyperpolarized, such as during sleep or anesthesia"**. We agree that spontaneous, thalamic post-inhibitory rebound activity is characteristic of the unconscious state. However, when the thalamic neurons are driven by a sensory stimulus, post-stimulus rebound activity can be observed in the awake subject. We added a paragraph making this clear to the discussion (lines 435-439): "While post-inhibitory rebound bursting activity of thalamic neurons is characteristic of the unconscious state, it can be observed in the auditory thalamus of awake, passively listening animals as well in the form of a post-stimulus burst [Cooke et al., 2020], even though at longer stimulus durations than used in the model presented here.".

Overall, we believe that our model has a solid neurobiological basis. However, we now highlight and discuss more prominently in the manuscript that the main data underlying the model originate from anesthetized subjects and we acknowledge that the potential translation of our insights to awake subjects may be limited by this fact. Nevertheless, we are confident that our results are relevant for a broad audience in the field of sensory processing.

Comment 3.2: The model does not include inhibitory neurons in cortex. Without such neurons, epileptic-like activity is expected to propagate in cortex due to the excitatory-to-excitatory connections within each layer. The authors did not respond to this criticism, and discuss properties of TC neurons instead. The authors need to examine under which conditions such epileptic-like state exists by examining the connections within later 4 and within layer 6. They need to clarify in which regime does they obtain SSA but no epileptic-like activity in cortex.

Response 3.2: Thanks to the reviewer for the comment. Our model employed synaptic depression as a surrogate for inhibitory neurons to prevent overexcitation in the cortex. Fig. 2 B and C shows the sensitivity of SSA in cortical L4, L6 and thalamus to the recovery time constant of synaptic connections within cortical L4 and L6, respectively. The effect of SSA is quantified by the SSA index (SI), where positive values indicate the effect's existence (please see the Data analysis subsection of the manuscript for detailed metrics descriptions, lines 716-738). In particular, setting the synaptic resources to recover extremely fast (on the order of 1 ms as highlighted in the grey shade), the depression effects of the synapses become negligible and the cortical network generates epileptic-like behavior in response to a transient peripheral stimulus (depicted in the left panel of Fig. 2A). In contrast, balanced cortical activity (right panel of Fig. 2A) occurs when recovery of the depressing synapses is slower than 10 ms, and specifically, robust SSA can be obtained in the regime of hundreds of milliseconds to seconds of the synaptic recovery time constant.

In addition, we investigated the dependence of both SSA and true deviance detection on the intra- and inter-columnar connection strength within L4 and within L6 by varying one parameter at a time and fixing the rest to their given values, as demonstrated in Fig. 2D-G. No epileptic-like activity is able to be induced in these scenarios. The more positive value of the context-specificity index (CSI) indicates the stronger effect of true deviance detection. For lateral connections (right panel of Fig. 2E and G), deviance-detecting effects no longer exist when their strength exceeds a certain critical value (0.07 for J^1_{L4} and 0.08 for J^1_{L6}). On the other hand, the operating ranges of the recurrent connections where the model demonstrates qualitatively similar deviant behaviors to the experimental observations are 2.1 to 2.3 for J^0_{L4} and 2.5 to 2.6 for J^0_{L6}. The relatively lower ratio of the operating range of recurrent connections to their given values indicates that the deviance-detecting effects in our model are more susceptible to recurrent than lateral cortical connections. We have supplemented the above experiments in the Material and methods section of the revised manuscript (lines 679-706).

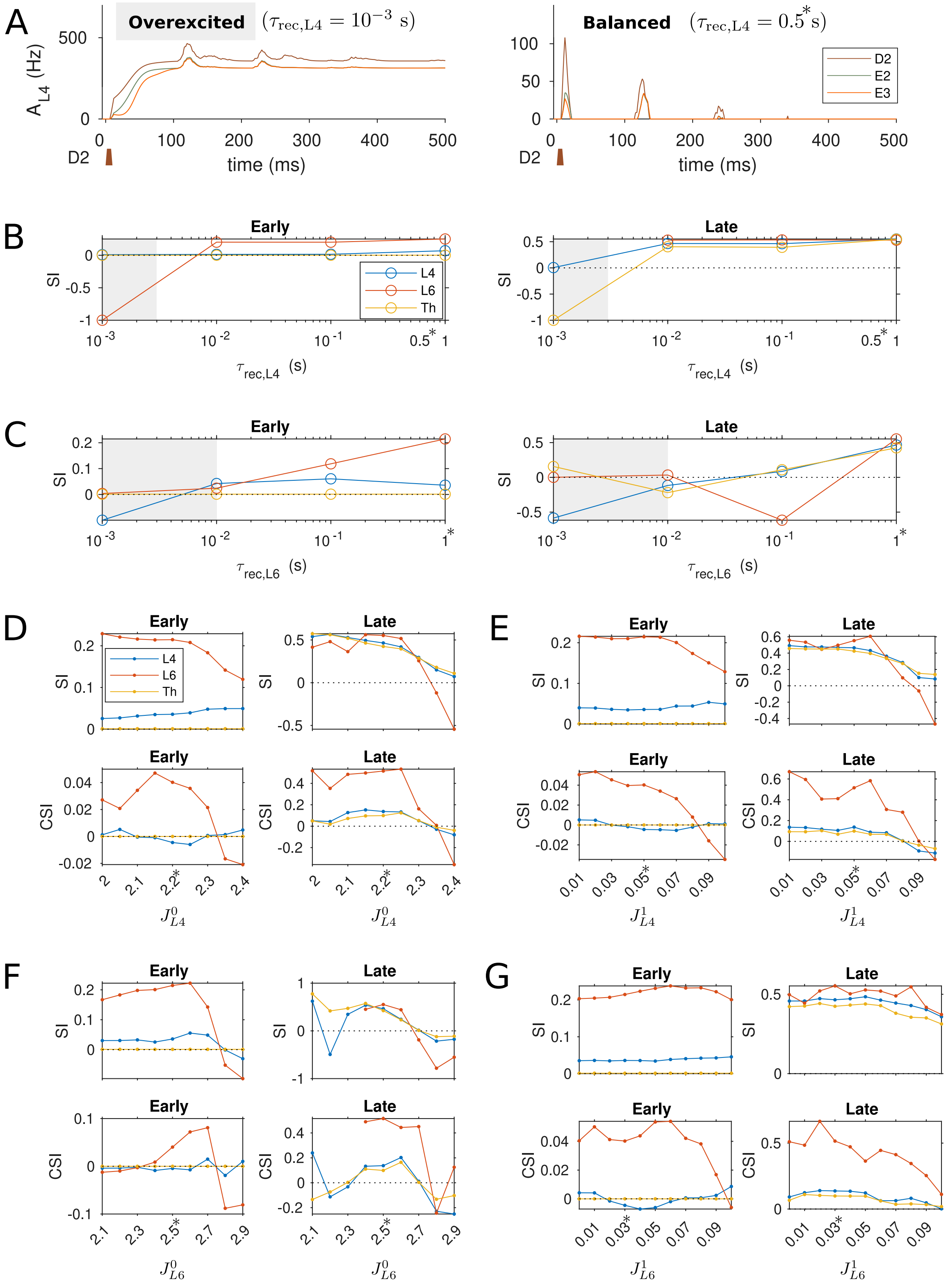

Figure 2: **Sensitivity of deviance-detecting effect and overexcitation to recovery time constants and strengths of depressing synapses in cortical L4 and L6. (A)** Overexcited and balanced L4 population activity evoked by a brief D2 whisker deflection (marked under the time axis) given fast and slow recovery timescales of L4 depressing synapses, respectively. The starred value in the title denotes the parameter's default value in Table 1 of the manuscript. **(B)** Dependence of SSA in L4, L6 and thalamus on the logarithmically-scaled recovery time constant of L4 synapses, $\tau_{\rm rec,L4}$. The regimes for overexcited and balanced cortical activity are respectively represented in grey and white backgrounds. **(C)** Same as **(B)**, but for L6 synapses, $\tau_{\rm rec,L6}$. **(D-G)** Dependence of SSA and true deviance detection on the intra- and inter-columnar connection strengths within L4, $J^0_{\rm L4}$, $J^1_{\rm L4}$, and L6, $J^0_{\rm L6}$, $J^1_{\rm L6}$. All responses fall into the regime of balanced activity when modifying these lateral and recurrent connections. The starred values under the horizontal axes represent the parameters' default values. The random seed of model simulation is kept fixed when changing the values of each connection parameter.

Comment 3.3: The authors admit that: "Indeed, parameters of thalamic neurons and their connectivity strength need to be finely tuned to demonstrate the decaying rhythmic bursting behavior". It is therefore unclear whether the model is robust. The authors need to examine the robustness of their results by modifying the strength of the thalamocortical or cortico-thalamic connections. They need to report in which regime decaying oscillations are obtained (such as in Fig. 3), in which regime the oscillations continue "forever" and do not decay, and in which regime there are no oscillations.

Response 3.3: We studied the sensitivity of SSA and true deviance detection to the cortico-thalamic and intra-thalamic connection strength, as well as regimes of different thalamic late activity.

Given different combinations of cortico-thalamic and intra-thalamic coupling strength, the thalamic circuit can trigger three late response patterns (see Fig. 3A), which are also reflected in cortical L4 due to the thalamocortical pathway. As can be seen in Fig. 3B-E, the deviance-detecting effect is more robust in the decaying oscillation regime than in the other two regimes. Particularly, we found that as the TC-to-RE synaptic conductances increases, the regime of no oscillation shrinks while the regime of sustained oscillation expands (Fig. 3B-D). This transition of regimes is likely because the RE cells become more excitable by the increased conductances from TC cells onto them and, in turn, provide more inhibition to TC cells. Consequently, more rebound bursts are triggered in TC cells, which repeats the cycle of firings of RE cells. Finally, we fixed the cortico-thalamic connection strength to its default value and examined the sensitivity of the results to the intra-thalamic coupling strength (Fig. 3E). As the TC-to-RE synaptic conductance increases, the response regime shifts from decaying oscillation to sustained oscillation, which weakens and even eliminates the deviance-detecting effect in the late responses. We now add the new experiments in lines 707-723 of the revised manuscript.

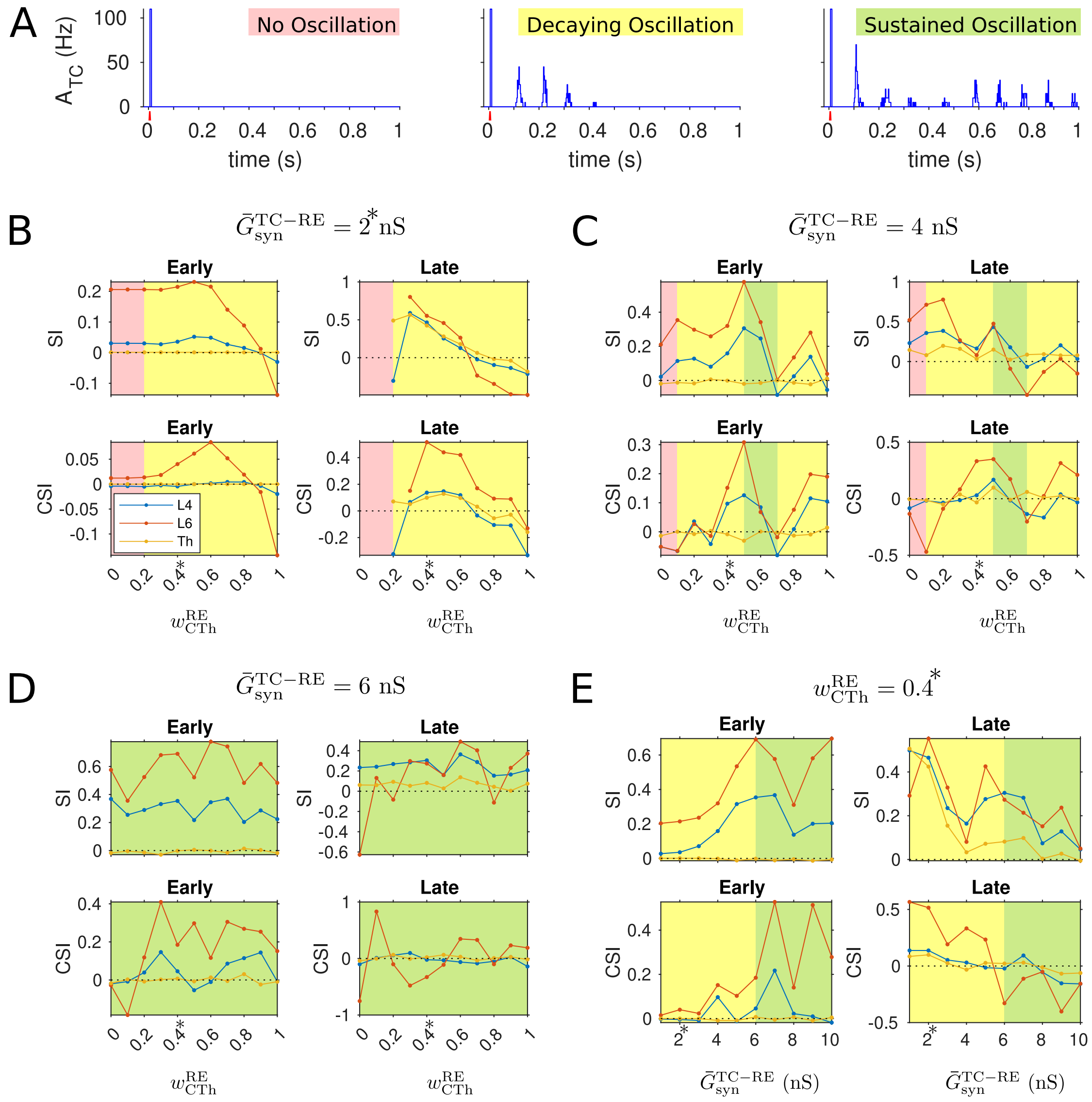

Figure 3: **Dependence of deviance-detecting effect and thalamic response patterns on the cortico-thalamic and intra-thalamic connection strength.** **(A)** Three late oscillation patterns (regimes) of thalamocortical cells can be elicited by a whisker deflection (marked in red under the time axis). **(B-D)** Sensitivity of SSA and true deviance detection in L4, L6 and thalamus to the strength of cortico-thalamic coupling on RE cells, w_{CTh}^{RE}, given different TC-to-RE synaptic conductances, $\bar{G}_{\text{syn}}^{\text{TC-RE}}$. Background colors denote one of the three regimes depicted in **(A)**. **(E)** Analogous to **(B-D)**, but by changing TC-to-RE synaptic conductances, $\bar{G}_{\text{syn}}^{\text{TC-RE}}$, with cortico-thalamic coupling strength, w_{CTh}^{RE}, fixed. The starred value denotes the parameter's default value.

Comment 3.4: The response to sequenced deviant is almost the same as the response to randomize deviant. The authors have not convinced that the small difference is not a result of a particular choice of parameters. Figure 6 should be removed.

Response 3.4: We have removed Fig. 6B and its corresponding paragraphs from the revised manuscript.

References

Cooke, J. E., Lee, J. J., Bartlett, E. L., Wang, X., and Bendor, D. (2020). Post-stimulatory activity in primate auditory cortex evoked by sensory stimulation during passive listening. *Scientific Report*, 10(1):13885.

Fanselow, E. E., Sameshima, K., Baccala, L. A., and Nicolelis, M. A. (2001). Thalamic bursting in rats during different awake behavioral states. *Proceedings of the National Academy of Sciences*, 98(26):15330–15335.

Marx, M. and Feldmeyer, D. (2013). Morphology and physiology of excitatory neurons in layer 6b of the somatosensory rat barrel cortex. *Cerebral Cortex*, 23(12):2803–2817.

Mease, R. A., Krieger, P., and Groh, A. (2014). Cortical control of adaptation and sensory relay mode in the thalamus. *Proceedings of the National Academy of Sciences*, 111(18):6798–6803.

Musall, S., Haiss, F., Weber, B., and von der Behrens, W. (2017). Deviant processing in the primary somatosensory cortex. *Cerebral Cortex*, 27(1):863–876.

Narayanan, R. T., Egger, R., Johnson, A. S., Mansvelder, H. D., Sakmann, B., de Kock, C. P. J., and Oberlaender, M. (2015). Beyond columnar organization: Cell type- and target layer-specific principles of horizontal axon projection patterns in rat vibrissal cortex. *Cerebral Cortex*.

Swadlow, H. A. and Gusev, A. G. (2001). The impact of'bursting'thalamic impulses at a neocortical synapse. *Nature neuroscience*, 4(4):402–408.

Whitmire, C. J., Waiblinger, C., Schwarz, C., and Stanley, G. B. (2016). Information coding through adaptive gating of synchronized thalamic bursting. *Cell reports*, 14(4):795–807.

Wright, N. C., Borden, P. Y., Liew, Y. J., Bolus, M. F., Stoy, W. M., Forest, C. R., and Stanley, G. B. (2021). Rapid cortical adaptation and the role of thalamic synchrony during wakefulness. *The Journal of Neuroscience*, 41(25):5421–5439.
