# Supplementary material for: Modelling novelty detection in the thalamocortical loop
Source: PLoS Comput Biol. 2023 May 15;19(5):e1009616. doi: 10.1371/journal.pcbi.1009616 (PMC10212104; doi:10.1371/journal.pcbi.1009616)
Supplement: Attachment — Submitted filename: Response to reviewers.pdf [file pcbi.1009616.s005.pdf]

Responses and Changes for PCOMPBIOL-D-21-02007R2: Modelling novelty detection in the thalamocortical loop

Chao Han, Gwendolyn English, Hannes P. Saal, Giacomo Indiveri, Aditya Gilra, Wolfger von der Behrens, Eleni Vasilaki

---

We would like to sincerely thank the editor and three anonymous reviewers for the valuable comments. We have addressed a remaining issue raised by reviewer 3. We had already removed the results panel 6B of Fig. 6 as requested in the earlier round, but had retained panel 6A which illustrated the protocols used for Fig. 7. Now, we have merged the same with Fig. 7, else on its own Fig 6A appeared irrelevant as pointed out by the reviewer. But we feel without that panel, it is difficult for the reader to interpret Figure 7. Please note that we have **highlighted** the changed text in red addressing the concerns in our revised manuscript. We hope that these is acceptable otherwise we are happy to accomodate any relevant changes or suggestions.

Email addresses: c.han@sheffield.ac.uk (Chao Han), e.vasilaki@sheffield.ac.uk (Eleni Vasilaki)

Responses to Comments from Reviewer 3

In the revised version, the model is presented as a model for anaesthetized somatosensory cortex. Whether this makes it less interesting, the existence of thalamic bursts is easier to justify based on experimental observations. Since the system can be less active for certain forms of anesthesia, it is more justified to neglect inhibition in cortex.

Comment 3.1: **The authors need to remove the remaining of Fig. 6. After panel 6B was removed, the remaining panel does not carry enough relevant information to be shown.**

Response 3.1: We thank the reviewer for the comment. In the previous round of revisions, the reviewer had requested the removal of Fig. 6 as the results were not convincing enough. Accordingly, we had removed panel 6B which contained the results, while retaining panel 6A as this also illustrated the protocols for Fig. 7. However, as the reviewer points out, this panel on its own seems irrelevant. Thus, we have merged it into Fig. 7, which ideally we should done already in the previous round. We think this will provide the reader with an illustrative understanding of the protocols for Fig. 7. The new combined figure now becomes Fig. 6 (see page 15 of the revised manuscript).
